# The Effect of Specific Soil Microorganisms on Soil Quality Parameters and Organic Matter Content for Cereal Production

**DOI:** 10.3390/plants10102000

**Published:** 2021-09-24

**Authors:** Arnoldas Jurys, Dalia Feizienė

**Affiliations:** Department of Plant Nutrition and Agroecology, Institute of Agriculture, Lithuanian Research Centre for Agriculture and Forestry, Instituto al. 1, LT-58344 Akademija, Lithuania; dalia.feiziene@lammc.lt

**Keywords:** soil organic carbon, plant-growth-promoting rhizobacteria, plant-growth-promoting fungi, microbial biodiversity

## Abstract

Soil chemical, biological and physical properties play important roles in soil quality and are related with increasing organic matter content, soil microbiological activity, plant nutrient content and availability. A new generation of soil amendments, containing specific soil microorganisms, are of great interest worldwide. Field experiments were carried out in 2018–2019 at the Institute of Agriculture, Lithuanian Research Centre for Agriculture and Forestry. The aim was to determine the impact of bio-products containing *Trichoderma reesei*, *Acinetobacter calcoaceticus* or *Bacillus megaterium*, and their mixtures on changes of soil organic carbon (SOC), soil respiration, and microbial biodiversity in loamy *Cambisol*. Under dry meteorological conditions, *Trichoderma reesei*, *Acinetobacter calcoaceticus* and *Bacillus megaterium* bio-products resulted an increase in SOC content, C/N ratio, humic to fulvic acid ratio, soil respiration, and microbial biodiversity. It is concluded that the use of a mixture of three microorganisms (*Trichoderma reesei* + *Acinetobacter calcoaceticus* + *Bacillus megaterium*) is the most promising bio-amendment under climate change. Future research is needed on different soil types and textures.

## 1. Introduction

Naturally, soil is a slowly renewable resource with a high degree of degradation and very low rate of regeneration [1]. Intensive agriculture leads to soil erosion, depletion of organic matter and other nutrients, which results in permanent soil degradation and significant productivity losses [2]. To maintain optimal yields and avoid soil depletion, it is necessary to protect soil from organic carbon and biodiversity losses, micronutrient imbalance, acidity, and salinity [3]. The use of microbial inoculants while developing sustainable agriculture techniques in chemical-based farming is one of the most promising alternatives these days [4]. Input of exogenous organic matter, such as straw or livestock manure, can enrich soil with the necessary elements and prevent soil organic matter (SOM) decomposition [5]. Soil quality parameters can be determined analyzing soil chemical, biological, and physical properties together [2,3]. To determine soil quality, indicators such as particulate organic matter (OM), active C, total N, microbial biomass, biological activities, enzymes, soil pH, cation exchange capacity, salinity, bulk density, amino sugar, and soil aggregation should be known [2,6]. Soil microorganisms consist mainly of primary decomposers that mineralize organic materials and release nutrients and energy for plants by enzyme-facilitated metabolic systems [2,6,7]. Plant growth and yield in natural environments depend on many interactions between plant roots, bacteria, and fungi [6,8]. Regarding the decomposition of organic residues, microbial communities can be broken down into fungal and bacterial groups, and research indicates that these groups function differently in the decomposition processes [9,10]. Bacterial decomposition pathways support high turnover rates of easily available substances, fungal-dominated decomposition pathways are slower and includes degradation of complex organic materials [6,9,10]. It is known that the main plant-beneficial rhizosphere microorganisms are mycorrhiza, rhizobia bacteria, PGPR (plant-growth-promoting rhizobacteria) such as *Pseudomonas* spp., *Bacillus* spp. or *Azospirillum* spp., and PGPF (plant-growth-promoting fungi) such as *Trichoderma* spp. and non-pathogenic *Fusarium* spp. strains [7,10,11]. PGPR and PGPF colonize plant roots and are responsible for plant defense, protecting them not only from various foliar pathogens but also from leaf-feeding insects [10,11].

In this research two strains of PGPR and 1 strain of PGPF were used. *Acinetobacter calcoaceticus*—a Gram-negative, rod-shaped (0.9–1.6 µm by 1.5–2.5 µm) in the intensive growth phase, aerobic soil bacterium belonging to the genus of *Acinetobacter*. In the growth phase it is normally grouped in pairs or chains. *Acinetobacter calcoaceticus* has no sporulation properties. The growth temperature mode is from 15 to 37 °C. *Bacillus* *megaterium* is a Gram-positive, rod-shaped (2.0–5.0 µm by 1.2–1.5 µm), mainly aerobic soil bacterium, belonging to the genus *Bacillus*. It is one of the largest known bacteria. *Bacillus megaterium* can form spores that can be ellipsoidal, spherical, central, paracentral or subterminal. Growth temperature depends on environment where it was extracted, and could be from 3 to 45 °C; the optimum is about 30 °C. Both bacteria are known for their phosphorus-releasing and nitrogen-fixing properties [12,13,14]. *Trichoderma reesei* is a filamentous fungus, widely distributed in places of soil and wood decomposition. This microscopic fungus is one of the main microorganisms used for the industrial production of biomass degrading enzymes [15,16]. Saprophytic fungus of the genus *Trichoderma* obtains its nutrients from dead and decaying plant matter. *Trichoderma reesei* is known as a plant cell wall degrader which efficiently produces enzymes which degrade plant cell wall compounds, such as cellulose, hemicellulose, and pectin [17]. Under special conditions after 1–3 weeks this fungi can form ascospores.

There is no literature concerning the influence and relationship of tillage with microorganisms. This study aims to determine the influence of specific soil microorganisms (PGPR and PGPF individually and in a complex) on soil organic carbon (SOC), soil respiration and biodiversity in loamy Cambisol under reduced tillage conditions.

We hypothesize that bio-products which contain *Trichoderma reesei*, *Acinetobacter calcoaceticus* or *Bacillus megaterium* and their mixtures will increase the SOC content, C/N ratio, humic to fulvic acid ratio, soil respiration and microbial biodiversity in loamy *Cambisol*.

The aim of research was to investigate the influence of new generation amendments on soil organic carbon content, soil vitality, and biodiversity.

## 2. Results

Microorganisms were selected from four different farming practices using selective media (Table 1). During microorganism isolation processes, the total number of microorganisms was not as relevant as the goal of finding specific microorganisms.

After screening for specific microorganisms and evaluating the possibility of nitrogen fixation and phosphorus solubilization, it was decided to continue field trials with microorganisms isolated from a chemical farm with five field crop rotations and natural meadows. From the chemical farm with five field crop rotations, two isolated microorganisms were selected. The isolated bacterial strain had very good phosphate solubilization properties and good nitrogen fixation properties. Due to phenotypic traits and formation of yellow pigment after 48 h of cultivation, it was thought to be *Bacillus megaterium*. Purified microscopic fungal culture had similar phenotypic traits to *Trichoderma* spp. The bacterial strain isolated from natural meadows had great phosphate solubilization and nitrogen fixation properties, and also grew faster than bacteria selected from the chemical farm.

Selected microorganism cultures were purified to a single colony before samples were sequenced and identified. For confirmation, results were double checked in public databases. The bacteria which was isolated from natural meadows was *Acinetobacter calcoaceticus* with a 99.94% match. As expected, samples from the chemical farm with five field crop rotations had a 99.99% match with *Bacillus megaterium*, and *Trichoderma reesei* had 100.00% similarity.

**Figure 1:**
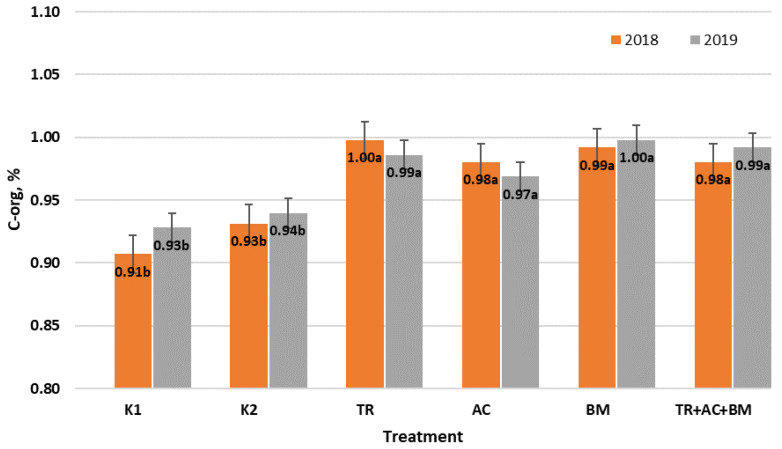
Average organic carbon concentration of soil during 2018–2019 field experiments. Different letters in blocks indicate significant differences in 2018 and 2019 (*p* < 0.05). K1—control 1, K2—control 2, TR—*Trichoderma reesei*, AC—*Acinetobacter calcoaceticus*, BM—*Bacillus megaterium*, TR + AC + BM—*Trichoderma reesei + Acinetobacter calcoaceticus + Bacillus megaterium*.

According to the SOC content, the soil of experimental fields could be classified as having a low SOC content. At the beginning of the experiment, SOC ranged from 0.91 to 0.93%. During 2018–2019 seasons (Figure 1), the average SOC content was higher in all treatments with microorganisms, compared to control 1 (K1) and control 2 (K2). In 2018, the highest SOC concentration had been in treatment 3 (*Trichoderma reesei*). This was 10.0% higher than in K1 and 7.1% higher than in K2. In 2019, the highest SOC was in treatment 5 (*Bacillus megaterium*), i.e., 7.5% higher compared to K1 and 6.2% higher compared to K2.

**Figure 2:**
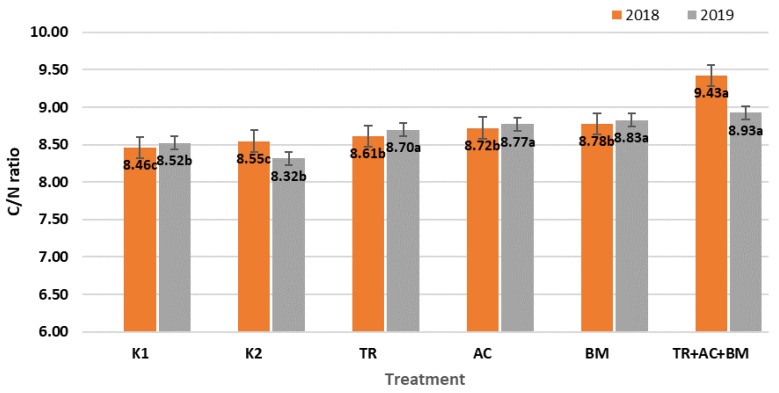
Average C/N ratio during 2018–2019 field experiments. The different letters in blocks indicate significant differences in 2018 and 2019 (*p* < 0.05). K1—control 1, K2—control 2, TR—*Trichoderma reesei*, AC—*Acinetobacter calcoaceticus*, BM—*Bacillus megaterium*, TR + AC + BM—*Trichoderma reesei + Acinetobacter calcoaceticus + Bacillus megaterium*.

The decomposition intensity of organic matter is best determined by the C/N ratio. In the 2018 period the C/N ratio (Figure 2) was 11.4% higher in treatment 6 (*Trichoderma reesei*, *Acinetobacter calcoaceticus*, *Bacillus megaterium*) compared to K1 and 10.3% higher compared to K2. In the 2019 period the C/N ratio (Figure 2) was highest in treatment 6; which was 4.7% higher than K1 and 7.3% than K2. The increased C/N ratio during the experimental period means that the ratio of mineralization/humification processes changed.

**Figure 3:**
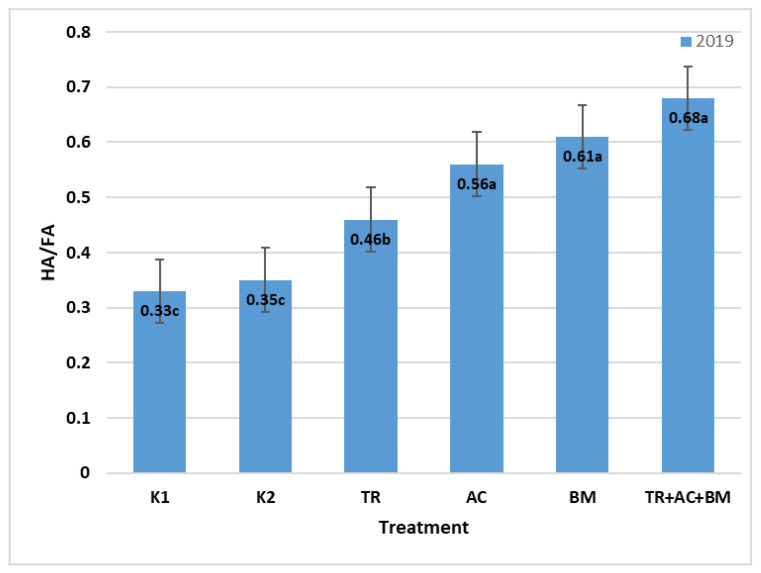
Average humic to fulvic acid ratio in 2019. The different letters in blocks indicate significant differences in 2019 (*p* < 0.05). K1—control 1, K2—control 2, TR—*Trichoderma reesei*, AC—*Acinetobacter calcoaceticus*, BM—*Bacillus megaterium*, TR + AC + BM—*Trichoderma reesei + Acinetobacter calcoaceticus + Bacillus megaterium*.

Soil humus, especially its stable fractions, is formed slowly, so this indicator does not fully reflect the humification processes of embedded organic matter. The high humus content and low humic and fulvic acid ratio indicate that this humus is not long-lasting and the humification processes in such soils were weak. The humic to fulvic acid ratio was measured in 2019. Highest humic to fulvic (Figure 3) ratio was in treatment 6 (*Trichoderma reesei*, *Acinetobacter calcoaceticus*, *Bacillus megaterium*)—106.1% higher than K1 and 94.3% higher than K2.

**Figure 4:**
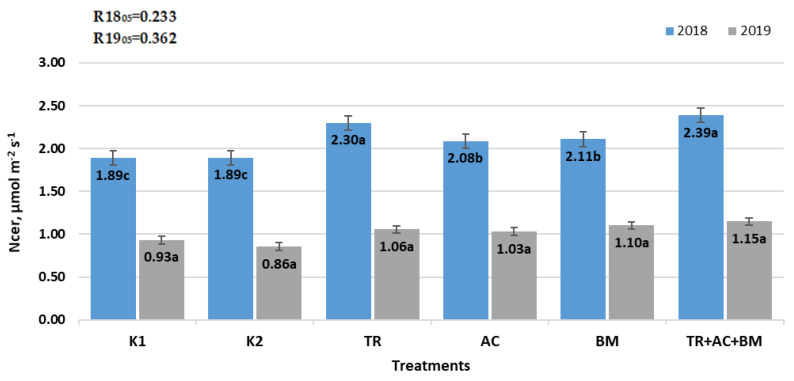
Average soil net CO_2_ exchange rate. The different letters in blocks indicate significant differences in 2018 and 2019 (*p* < 0.05). K1—control 1, K2—control 2, TR—*Trichoderma reesei*, AC—*Acinetobacter calcoaceticus*, BM—*Bacillus megaterium*, TR + AC + BM—*Trichoderma reesei + Acinetobacter calcoaceticus + Bacillus megaterium*.

During the period of experiment, the soil NCER mean (Figure 4) was highest in treatment 6 (*Trichoderma reesei* + *Acinetobacter calcoaceticus* + *Bacillus megaterium*). In 2018, the NCER mean was higher by 26.3%, compared to both K1 and K2. In 2019, the NCER mean was 23.4% higher than in K1 and 33.3% higher than K2.

**Figure 5:**
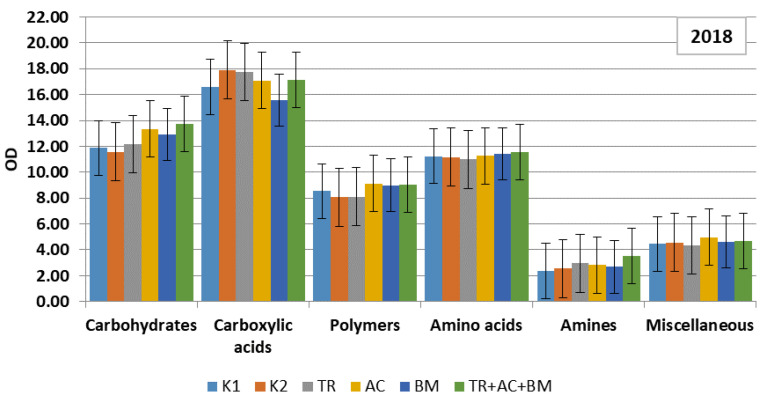
Level of consumption of substrates by microbial communities of soil in 2018. On the y axis OD—optical density, on the x axis, carbon sources. K1—control 1, K2—control 2, TR—*Trichoderma reesei*, AC—*Acinetobacter calcoaceticus*, BM—*Bacillus megaterium*, TR + AC + BM—*Trichoderma reesei + Acinetobacter calcoaceticus + Bacillus megaterium*.

**Figure 6:**
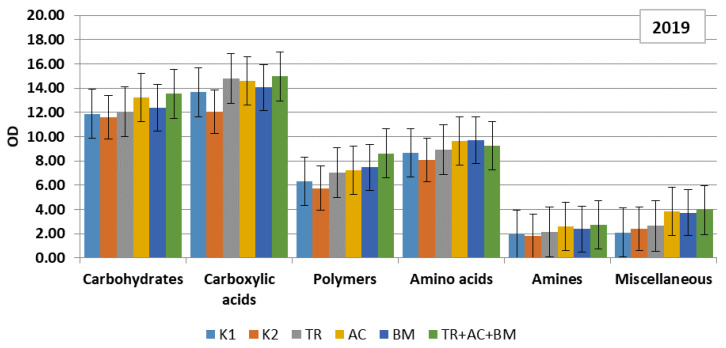
Level of consumption of substrates by microbial communities of soil in 2019. On the y axis OD—optical density, on the x axis, carbon sources. K1—control 1, K2—control 2, TR—*Trichoderma reesei*, AC—*Acinetobacter calcoaceticus*, BM—*Bacillus megaterium*, TR + AC + BM—*Trichoderma reesei + Acinetobacter calcoaceticus + Bacillus megaterium*.

To compare the strategy of substrate consumption by the soil microbiomes, the matters were contained in a Biolog EcoPlate and were classified into six main groups: carbohydrates, carboxylic acids, polymers, amino acids, amines, amides and miscellaneous. Data revealed that in both dry experimental years carboxylic acids were dominating. In 2018 they consisted of 28–32% and in 2019, 28–31% of all substrate C sources (Figure 5 and Figure 6). The amount of carbohydrates was 21–23% and 25–28%, amino acids 19–20% and 17–20%, polymers 14–16%, amines 4–6% and 4–5%, and miscellaneous 8% and 5–8%, respectively.

In the first dry year, the influence of soil microbiological amendments on C sources of the soil substrate were insignificant (Figure 5). In the next dry year, all products caused significantly higher optical density (OD) values in each substrate category, compared to both controls. The most significant effect was revealed in the combined application of treatment 6 (*Trichoderma reesei*, *Acinetobacter calcoaceticus*, *Bacillus megaterium*) (Figure 6). High levels of carbohydrates and carboxylic acids in substrate consumption may relate to the complexity of these substrates.

Principle Components Analysis (PCA) of average well color development (AWCD) data revealed that during the first 4 days of the investigation, average well color development (AWCD) had grown in all selected samples. This index shows that microbial community metabolic activity was high, which may relate to carbon substrates. In 2018, on the 4th day of incubation, the highest AWCD index was found in treatment 6 (*Trichoderma reesei*, *Acinetobacter calcoaceticus*, *Bacillus megaterium*), which was 21–22% higher than in K1 and K2. Meanwhile, treatments 3–5 differed from controls K1 and K2 by only 3–8%.

**Figure 7:**
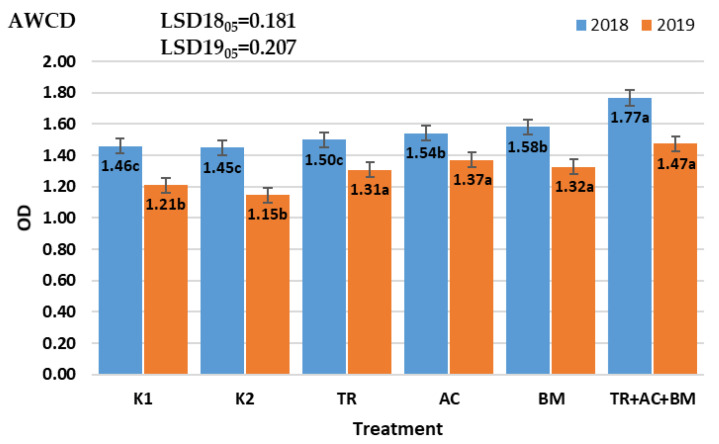
Influence of microbiological amendments on AWCD. The different letters in blocks indicate significant differences in 2018 and 2019 (*p* < 0.05). K1—control 1, K2—control 2, TR—*Trichoderma reesei*, AC—*Acinetobacter calcoaceticus*, BM—*Bacillus megaterium*, TR + AC + BM—*Trichoderma reesei + Acinetobacter calcoaceticus + Bacillus megaterium*.

In 2019, on the 4th day of incubation, the highest AWCD index was also found in treatment 6 (*Trichoderma reesei*, *Acinetobacter calcoaceticus*, *Bacillus megaterium*), which was 22–28% higher than in K1 and K2 treatments. Treatments 3–5 differed by only 8–13% from controls K1 and K2 (Figure 7).

**Figure 8:**
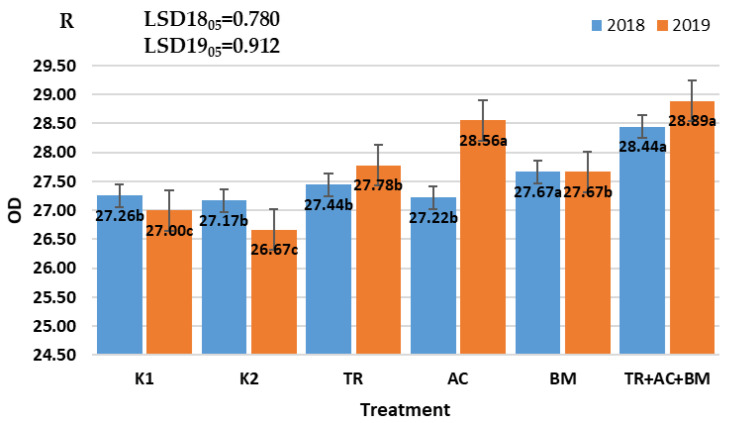
Influence of microbiological amendments on soil R index. The different letters in blocks indicate significant differences in 2018 and 2019 (*p* < 0.05). K1—control 1, K2—control 2, TR—*Trichoderma reesei*, AC—*Acinetobacter calcoaceticus*, BM—*Bacillus megaterium*, TR + AC + BM—*Trichoderma reesei + Acinetobacter calcoaceticus + Bacillus megaterium*.

The richness (R) index showed that the microbial diversity varied depending on the type of soil management technique, and generally increased when the combined application of microbiological products in treatment 6 (*Trichoderma reesei*, *Acinetobacter calcoaceticus*, *Bacillus megaterium*) were used (Figure 8). Microbial richness varied significantly (*p* < 0.05) in both experimental years. In 2018 the highest R value was determined in treatment 6 (*Trichoderma reesei*, *Acinetobacter calcoaceticus*, *Bacillus megaterium*) and was 4–5% higher than in K1 and K2 treatments. Meanwhile, differences from controls K1 and K2 and treatments 3–5 were insignificant. In 2019, the highest R value was determined in treatments 4 (*Acinetobacter calcoaceticus*) and 6 (*Trichoderma reesei*, *Acinetobacter calcoaceticus*, *Bacillus megaterium*). The R index was 6–7% and 7–8% higher, respectively, than in K1 and K2. The correlation between AWCD and R indices was very high (r = 0.91–0.95). Increased values of ACWD and R is likely related with SOC increase.

From an agronomic point of view, the most valuable aggregates are those that do not decompose when exposed to water and can retain their stability for a long time. Overall, the stability of soil aggregates in the experimental field was good.

**Figure 9:**
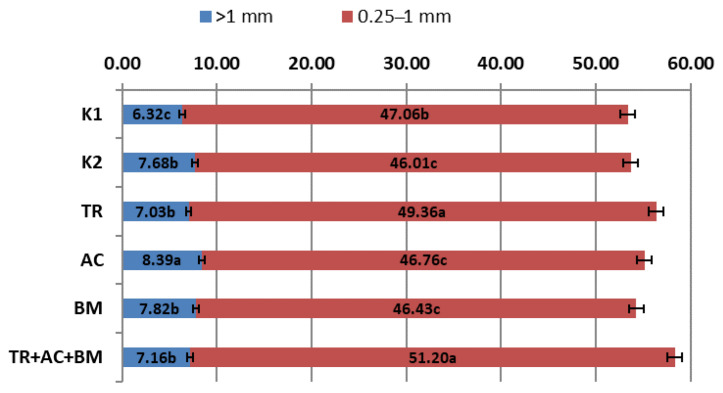
Stability of soil aggregates in the 0–10 cm layer. The different letters in blocks indicate significant differences in 2018 and 2019 (*p* < 0.05). K1—control 1, K2—control 2, TR—*Trichoderma reesei*, AC—*Acinetobacter calcoaceticus*, BM—*Bacillus megaterium*, TR + AC + BM—*Trichoderma reesei + Acinetobacter calcoaceticus + Bacillus megaterium*.

**Figure 10:**
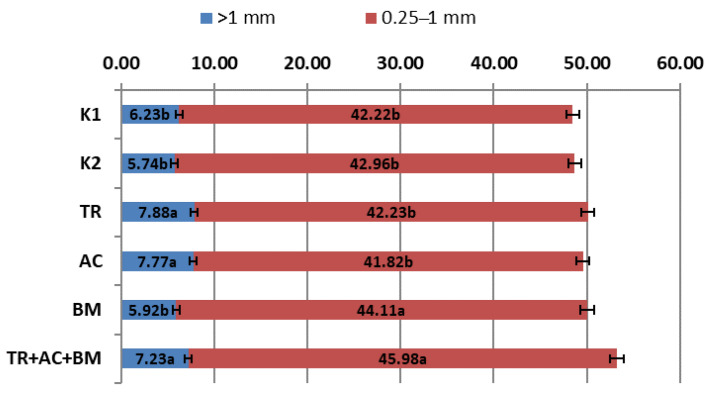
Stability of soil aggregates in the 10–20 cm layer. The different letters in blocks indicate significant differences in 2018 and 2019 (*p* < 0.05). K1—control 1, K2—control 2, TR—*Trichoderma reesei*, AC—*Acinetobacter calcoaceticus*, BM—*Bacillus megaterium*, TR + AC + BM—*Trichoderma reesei + Acinetobacter calcoaceticus + Bacillus megaterium*.

The stability of all aggregates in the top arm layer (0–10 cm) were higher than 53% (Figure 9) and in the lower arm layer (10–20 cm) (Figure 10) even reached 48%. Stable aggregates (0.25–1 mm) in the top arm layer (0–10 cm) on average reached 47.8% and 43.22% in the lower arm layer (10–20 cm). Aggregates of size >1 mm was 7.40% in the top arm layer (0–10 cm) and reached 6.80% in the lower arm layer. These results show that the physical soil structure was in good condition. During this experiment the differences of aggregate stability depended on the preparations used. In the top soil layer (0–10 cm), treatments 3 (*Trichoderma reesei*) and 6 (*Trichoderma reesei*, *Acinetobacter calcoaceticus*, *Bacillus megaterium*) were 4.9–8.8% higher than in K1 and 7.3% and 11.3% than K2. In the lower arm layer (10–20 cm), treatments 5 (*Bacillus megaterium*) and 6 (*Trichoderma reesei*, *Acinetobacter calcoaceticus*, *Bacillus megaterium*) showed the best results, which were 4.5% and 8.9% higher than in K1 and 2.7% and 7.0% than K2.

## 3. Discussion

Climate change and production intensification conditioned significant changes in the soil environment. Poor agricultural management practices and conditions granted by water, wind, or tillage erosion cause notable loses of the valuable topsoil layer [15]. The whole complex has a negative impact on soil microbial activity [15]. Therefore, worldwide there has been increased interest in the application of microbial amendments that provide natural benefits for soil and crops. Under commercial field conditions, microbial amendments can play an important role in environmental and human health protection [16].

Securing and improving soil quality is the main challenges to face when humanity is dealing with climate change. Considering the importance of the microbiota in soil ecosystems, the exploitation of microbial activity could have a great impact on soil properties improvement. The application of microbial amendments could improve usage of plant nutrients, reduce their release into soil and water, and consequently reduce negative effects to the environment [17].

The agrosector has used several strategies to rebuild the soil fertility. Most prevalent are livestock manure (fresh, composted, or solid fractions) from various species (cattle, hogs, poultry) or other amendments derived from agriculture including crop residues (straw, legumes) or spent mushroom compost [5,15]. These substances are also processed by specific soil microorganisms. When using these materials, it is important to ensure that efficient microorganisms dominate during the degradation processes, because this may subsequently have an impact on the biodiversity of microorganisms in the future [4,15,18].

Our results obtained are in line with other published data. We found positive effects of microbiological soil amendments on Cambisol properties. Bio-products with *Trichoderma reesei*, *Acinetobacter calcoaceticus* and *Bacillus megaterium* caused increases in the SOC content, C/N ratio, humic to fulvic acid ratio, and improved soil vitality by increasing soil respiration. We suppose that such results were governed by changes in soil physical properties. Pore structure, soil aggregate stability and water retention are under investigation in our experiment. Similar results were reported from sodic soils [19].

Bio-products act through a complex mechanism including plant roots, nitrogen fixation, P-solubilization, excretion of phytohormones, production of substances suppressing phytopathogens, guarding plants from abiotic and biotic stresses and detoxification of belowground pollutants, etc. [20,21,22,23]. Therefore, we think that the new generation of microbiological products could be advantageous under intensive farming conditions in commercial agriculture.

## 4. Materials and Methods

### 4.1. Site and Soil Description

The field experiments were conducted in 2018–2019 at the Institute of Agriculture, Lithuanian Research Centre for Agriculture and Forestry (55°23′50″ N and 23°51′40″ E). The prevailing soil was Endocalcari-Epihypogleyic Cambisol (CMg-n-w-can). The composition of soil aggregates was 52–54% sand (2.0–0.05 mm), 29–32% silt (0.05–0.002 mm) and 14–19% clay (<0.002 mm); soil was loam. Research scheme and product applications are described in Table 2. During 2018–2019 two crops were grown: winter wheat (*Triticum aestivum* L.) cv. Ada and spring barley (*Hordeum vulgare* L.) cv. Luokė. A field experiment was set up in four replications. The gross plot size and net harvested area of each individual treatment was 30.0 and 22.0 m^2^, respectively.

### 4.2. Soil Sampling and Analysis

Total nitrogen (N_tot_) and total carbon (C_tot_ for estimation of C/N ratio) were analyzed by the dry combustion method using a CNS autoanalyser Vario EL III (Elementar Analy-sensystem GmbH, Germany) [21,24]. The closed chamber method was applied to quantify the total (autotrophic + heterotrophic) soil surface net CO_2_ exchange rate (NCER). CO_2_ fluxes were measured five times during each crop growing season, each measurement was made between 10 a.m. and 12 a.m. using a portable infrared CO_2_ analyzer (IRGA) attached to a data logger (LcSRS-1000; ADC BioScientific Ltd., UK). In each treatment, the collar was inserted to 7.0 cm soil depth; the chamber hood was placed on the collar for 5 min until results were recorded in the data logger. Measurements were made with four replications. Expression of soil surface net CO_2_ exchange rate:

(1) NCER=u(−Δc)

where u is the molar flow of air per square meter of soil (mol m^−2^ s^−1^) and Δc is difference in CO_2_ concentration through soil hood, dilution corrected (μmol mol^−1^).

Humic acid and fulvic acid were determined according to the “Agricultural Chemical Analysis, Method 5.4. Cabi Publishing, 2002” gravimetric and spectroscopic methods.

Substrate utilization potential as average well color development (AWCD) and the R index of the soil microbial community were determined according to the community level physiological profiles method using Biolog EcoPlates [22,25].

Soil water aggregate stability was determined by the wet sewing method with a Eijkelkamp Agriseerch Equipment apparatus (the Netherlands). Soil samples were taken at randomly selected sampling points at two places of each individual plot from the 0–10 and 10–20 cm layers (4 replicates per each layer for each treatment).

### 4.3. Soil Samples for Microorganism Extraction

Soil samples for microorganism extraction were collected from chemical farms with poor crop rotation, chemical farms with 5 field crop rotations, ecological farms, and natural meadows in the territory of the Republic of Lithuania. In each area, samples were collected from 5 different points with selected sites at a depth of 0–15 cm. The samples were mixed well to represent a single sample. After mixing, the sample was weighed for microbiological analysis, an air-dried sample was used to determine the pH (KCl). The pH value was measured for each sample.

### 4.4. Isolation of Microorganisms

Isolation of phosphorus-releasing/solubilizing microorganisms was routinely screened by a plate assay method using Pikovskaya (PVK) agar. The PVK medium was used for pre-selection (Table 3). Microorganisms showing the best results were transferred to the National Botanical Research Institute’s (NBRIY) (Table 3) phosphate growth medium to avoid the yeast extract effect [26,27].

Phosphorus-releasing/solubilizing microorganisms were isolated from each sample by serial dilution and spread plate method. First, 10 grams (10 ± 0.2 g) of soil sample was dispersed in 90 mL of autoclaved 0.9 % saline and mixed with a magnetic stirrer for 30 min. Serial dilutions to 10^−5^ were made for each soil sample, 0.1ml of each dilution was spread on Pikovskaya’s agar medium containing insoluble tricalcium phosphate and incubated at 30 °C for 3 days. The suitable colony counting range was 25–250 colonies per plate. Colonies showing halo zones were picked and purified [26,27]. Colonies of microorganisms that were able to form a halo zone were transferred to a new Petri dish with selective medium to check that the solubilization was not due to the influence of the surrounding microorganisms. The purified monoculture was transferred again to a selective medium to check whether the microorganism had not lost its phosphorus-releasing properties.

For isolation and preselection of nitrogen fixing bacteria Jensen’s Medium was used (Table 4). First, 10 grams (10 ± 0.2 g) of soil sample was dispersed in 90 mL of autoclaved 0.9% saline and mixed with magnetic stirrer for 30 min. Serial dilutions to 10^−5^ were made for each soil sample, 0.1 ml of each dilution was spread on Jensen’s Medium and incubated at 30 °C for 3 days. Bacteria able to grow on a nitrogen-free medium were picked and purified. The suitable colony counting range was 25–250 colonies per plate.

For isolation of microscopic fungi Sabouraud Dextrose Agar with Chloramphenicol was used (Table 5) [28]. Ten grams (10 ± 0.2 g) of soil sample was dispersed in 90 mL of autoclaved 0.9 % saline and mixed with a magnetic stirrer for 30 min. Serial dilutions to 10^−4^ were made for each soil sample, 0.1 ml of each dilution was spread on Sabouraud Dextrose Agar with Chloramphenicol and incubated at 25 °C for up to five days. The suitable colony counting range was 20–100 colonies per plate.

### 4.5. Microbial Identification

The culture of the selected microorganisms was purified to single colonies and transferred to petri dishes with Nutrient Broth medium for bacteria and Potato Dextrose agar for fungi. Prepared samples for identification of microorganisms were sent to BaseClear laboratory for the detection of samples. The goal was to identify bacterial and fungal samples using the validated MicroSEQ^®^ system from Applied Biosystems. This automated system is based on PCR amplification and DNA sequencing of bacterial (16S rDNA) or fungal (D2-LSU rRNA) genes. These genes are highly conserved among microbial species and allow for simple and fast identification.

After DNA lysis of the sample, the 16S ribosomal DNA (bacterial samples) or the D2-LSU gene (fungal samples) was amplified by PCR. PCR is then followed by a sequencing step. Samples were detected and analyzed using capillary electrophoresis and results were analyzed against the validated MicroSEQ library. The analysis was carried out according to the manufacturer’s specifications. For confirmation of results, public databases were double checked.

### 4.6. Statistical Analysis

The data were analyzed using software STATISTICA Base (version 6). One-way analysis of variance (ANOVA) was applied to evaluate the significance of differences between the means of the experimental data. The data were compared using Fisher’s least significant difference (LSD) test at the probability levels *p* < 0.05 and *p* < 0.01. Principal component analysis was performed.

## 5. Conclusions

Under drought conditions, bio-products with *Trichoderma reesei*, *Acinetobacter calcoaceticus* and *Bacillus megaterium* caused increases in SOC content, aggregate stability, C/N ratio, humic to fulvic acids ratio. All tested bio-products caused an increase in soil respiration. The highest respiration was observed in soil after application of a mixture with three products: *Trichoderma reesei* + *Acinetobacter calcoaceticus* + *Bacillus megaterium*. This was 23.7–26.5% higher compared to soil respiration in crop growing technologies without bio-products. The highest AWCD index and R index was found in treatments after application with a mixture of three products: *Trichoderma reesei* + *Acinetobacter calcoaceticus* + *Bacillus megaterium*.

The bio-amendments investigated exhibited potential value as means for soil viability and sustainability under climate change conditions in *Cambisols*. Future research is needed on different soil types and textures.

## Figures and Tables

**Table 1:** Quantification of soil suspension, screening of bacteria and fungi-specific properties.

	Chemical Farm with Poor Crop Rotation	Chemical Farm with 5 Field Crop Rotations	Ecological Farm	Natural Meadows
Total amount of phosphorus solubilizing bacteria CFU/g	4.1 × 10^5^	6.2 × 10^6^	5.3 × 10^5^	2.8 × 10^5^
Total amount of nitrogen fixing bacteria CFU/g	3.1 × 10^6^	8.1 × 10^6^	3.8 × 10^5^	3.1 × 10^5^
Total amount of fungi CFU/g	2.7 × 10^4^	2.5 × 10^5^	2.1 × 10^4^	3.2 × 10^4^
Sample pH	7.72	7.31	6.91	7.91

**Table 2:** Research design used the following doses of microorganisms: *Trichoderma reesei*—100 mL/ha, *Bacillus megaterium*—100 mL/ha, *Acinetobacter calcoaceticus*—100 mL/ha, ammonium nitrate—10 kg N/t residues.

Treatments	Abbreviations	Application of Bioproducts	
		After Harvesting—For Decomposition of Organic Residues	After Germination BBCH12–15
Control 1	K1	-	-
Control 2	K2	ammonium nitrate	-
*Trichoderma reesei*	TR	*Trichoderma reesei*	
*Acinetobacter calcoaceticus*	AC	-	*Acinetobacter calcoaceticus*
*Bacillus megaterium*	BM	-	*Bacillus megaterium*
*Trichoderma reesei* + *Acinetobacter calcoaceticus* + *Bacillus megaterium*	TR + AC + BM	*Trichoderma reesei*	*Acinetobacter calcoaceticus* + *Bacillus megaterium*

**Table 3:** Composition of media used for phosphorus solubilizing microorganisms g/L.

Pikovskaya (PVK) Agar		National Botanical Research Institute’s Phosphate Growth Medium (NBRIY)
Glucose	10.0	10.0
Yeast Extract	0.5	-
(NH_4_)_2_SO_4_	0.5	0.5
MgSO_4_·7H_2_O	0.1	0.1
Ca_3_(PO_4_)_2_	5.0	5.0
NaCl	0.2	0.2
KCl	0.2	0.2
MnSO_4_·2H_2_O	0.002	0.002
FeSO_4_·7H_2_O	0.002	0.002
Agar	15.0	15.0

**Table 4:** Composition of Jensen’s Medium g/L.

Sucrose	20.0
K_2_HPO_4_	1.0
MgSO_4_	0.5
NaCl	0.5
FeSO_4_	0.1
Na_2_MoO_4_	0.005
CaCO_3_	2.0
Agar	15.0

**Table 5:** Composition of Sabouraud Dextrose Agar with Chloramphenicol g/L.

Glucose	40.0
Peptone	10.0
Chloramphenicol	0.05
Agar	15.0
